# Differential Responses to UV-A Stress Recorded in Carotenogenic Microalgae *Haematococcus rubicundus*, *Bracteacoccus aggregatus*, and *Deasonia* sp.

**DOI:** 10.3390/plants11111431

**Published:** 2022-05-27

**Authors:** Konstantin Chekanov, Karina Shibzukhova, Elena Lobakova, Alexei Solovchenko

**Affiliations:** Department of Bioengineering, Faculty of Biology, Lomonosov Moscow State University, 1-12 Leninskie Gory, 119192 Moscow, Russia; shibzuhovaka@my.msu.ru (K.S.); elena.lobakova@gmail.com (E.L.); solovchenko@mail.bio.msu.ru (A.S.)

**Keywords:** photoprotection, UV-A, microalgae, *Bracteacoccus*, *Haematococcus*, carotenoids

## Abstract

UV-A is the main ultraviolet component of natural (solar) radiation. Despite it, its effect on phototrophs is studied less than UV-B. Effects of UV-A on photosynthetic apparatus of three carotenoid-producing microalgae were elucidated. Photosynthetic activity was studied using chlorophyll fluorescence analysis. Cell extracts were evaluated by absorbance spectroscopy. On the one hand, there were some common features of three strains. In all cases the changes involved PSII primary photochemistry and antennae size. All strains accumulated UV-absorbing polar compounds. On the other hand, some responses were different. Upregulation of non-photochemical quenching was observed only in *B. aggregatus* BM5/15, whereas in other cases its level was low. *H. rubicundus* BM7/13 and *Deasonia* sp. NAMSU 934/2 accumulated secondary carotenoids, whereas *B. aggregatus* BM5/15 accumulated primary ones. Microscopic features of the cultures were also different. *H. rubicundus* BM7/13 and *Deasonia* sp. NAMSU 934/2 were represented mostly by solitaire cells or small cell clusters, lacking their green color; the cells of *B. aggregatus* BM5/15 formed aggregates from green cells. Cell aggregation could be considered as an additional UV-protecting mechanism. Finally, the strains differed by their viability. *B. aggregatus* BM5/15 was most resistant to UV-A, whereas massive cell death was observed in two other cultures.

## 1. Introduction

Solar radiation is the primary source of energy for the biosphere. In the process of photosynthesis, it is captured and converted into energy-rich metabolites and reducing power. Although the photoassimilates are required for fixation of inorganic carbon by photoautotrophic organisms such as higher plants and (micro)algae, excessively captured light energy can kill the phototrophic cell. Photosynthetic apparatus (PSA) is the site of light energy assimilation in the cell and hence the main target of the photodestruction [1]. The spectrum of the sunlight covers the whole visible, infrared, and UV ranges. The latter contains high-energy photons mediating the damage caused by unattenuated solar radiation. Although the spectral quality of solar radiation undergoes annual and diurnal variations and affected by weather conditions and geographical location, it commonly includes UV-A radiation [2,3]. Near the surface of the Earth, solar spectrum includes 8–9% UV including 6.3% UV-A (320–400 nm) and 1.5% UV-B (280–320 nm) [2,3].

Mechanisms of destructive effects of UV-B are relatively well understood. UV-B quanta are absorbed by aromatic rings, S-S bonds, and peptide bonds of biomolecules. UV-B induces damage to nucleic acids and proteins, facilitates the generation of reactive oxygen species (ROS) in the presence of photosensitizers [2,3,4,5,6,7,8]. Despite a larger proportion of UV-A in solar spectrum, molecular basis of its effect on phototrophic cell are less elucidated [1]. Typical of UV-A is its bi-lateral effect on photoautotrophs: low and moderate doses stimulate photosynthesis and growth [1,2,5,9,10,11]. Thus, UV-A treatment leads to an increase in the energy transfer to photosystem (PS) I [10]. It modulates photoprotection and stimulates antioxidant systems [9], increases quantum efficiency of PSII and augments the cell metabolism [11]. The growth-promoting effect of UV-A is not accompanied by an increase in chlorophyll (Chl) content in the autotrophically cultivated cells; it is ascribed to an increase in PSA efficiency rather than to the PAR absorption capacity [11]. Some phototrophs can utilize UV-A for carbon assimilation even in the absence of visible light [12]. Positive effect of UV-A is also attributed to inhibition of viruses and bacteria infecting photosynthetic organisms, especially microalgae [13]. On the other hand, high UV-A doses can damage photosynthetic cell via mechanisms [14]. UV-A is highly damaging for photosynthesis [9]. Its primary target is PSII [9,13] including both donor and acceptor side of PSII reaction centers (RC) [9]. UV-A irradiation can also damage cytoskeleton [2] and facilitate ROS production via photosensitization of UV-A absorbing pigments [2]. N′-Formylkynurenine, an intermediate of tryptophan catabolism, reacts with nucleic acids after absorbing of UV-A quanta [2].

Protection of a photosynthetic cell against UV damage includes enzymatic systems for DNA reparation, chemical reaction of glutathione and ascorbate toward ROS neutralization, non-photochemical quenching of the excited Chl states, optic shielding by sunscreens such as phenolics, mycosporine-like amino acids (MAAs) and carotenoids [2,4,5,6,7,15,16,17]. Particularly, photolyases are activated by radiation in the range of 370–450 nm [2]. MAAs and carotenoids absorb radiation in the UV-A region of the spectra [4,5,6,17,18,19,20]. UV-A-protective mechanisms also include oligomerization of PSII RC and LHCII [10]. In plants, UV-A induces transcription of a certain group of genes, especially those involved in flavonoid biosynthesis [2]. UV-A activates signaling pathways in the cell [2]: phytochrome B (and probably other phytochromes) are involved in the protection from UV-A radiation [21].

Most of data on the effect of UV on PSA were obtained from the experiments with model organisms, such us *Pisum sativum*, *Spinacia oleracea*, *Arabidopsis thaliana*, or *Chlorella* spp. [2,3,9,10,15,19,21]. There are three main experimental strategies to study UV effect on phototrophs: outdoors culturing with natural light, outdoor culturing with addition of UV and culturing in artificial controlled conditions [8]. Latest include experiments on whole plants as well as plant organs, cell cultures and isolated plastids [2,8,9,10,11,15]. Some works were carried out on the cultures of unicellular algae. Changes in their pigment composition were evaluated by common approaches of absorbance spectroscopy in the visible range [2,6,10,11,12,14,19,22,23,24]. Valuable information on primary photochemistry was obtained by the analysis of chlorophyll fluorescence kinetics, O_2_ emission and CO_2_ assimilation rates [1,9,10,11,13,14,15,19,21,22,23,24,25]. Some data were obtained with the help of studies of thermoluminescence [9] and low-temperature fluorescence spectra [10], electron paramagnetic resonance spectroscopy [14], D1 protein immunoblotting [14], analysis of expression of genes of UV response [21,25], etc. HPLC or HPLC-MS are applied for separation and identification of secondary metabolites and phytohormones in response to UV [2,4,6,11,22,25]. Electron microscopy provides some important data on UV response of cell fine structure [2,3,15,22]. Light microscopic observations of cells is important part of works with unicellular algae (microalgae) [11,15]. The approaches attempted to study algal life cycle also have been reported [24,26,27]. In the macrophytic red algae *Mastocarpus stellatus* and *Chondrus crispus* an effect of UV-A and UV-B on germination and carpospores viability, as well as on PSA was shown [24]. These data were confirmed by standard statistical methods, such as multiple analysis of variance and/or repeated measures analysis of variance [24]. Metabolomic and transcriptomic data constitute new frontiers of the field of the studies of UV on photosynthesis. For the haptophyte *Isochrysis galbana* RNA-seq data were generated to analyze differential gene expression in response to natural UV [25]. The effect of UV on the expression of genes of diverse metabolic pathways and photosynthesis related genes was shown by statistical analysis of digital gene expression data using a model based on the negative binomial distribution with *p*-values corrected to control the false discovery rates [25]. At the metabolic level UV affects the content of Krebs cycle acids and the fractions of different groups of membrane lipids [25]. It should be noted that most of data were obtained either for UV-B or natural/mixed UV. Hence, collection of new information about the effect of UV-A on photosynthesis is a topical current issue.

Freshwater, aeroterrestrial, and soil microalgae dwell in the habitat characterized by adverse environmental conditions, such as high light, sharp temperature changes, and desiccation [16,19,28]. Accumulation of secondary carotenoids (the carotenoids structurally and functionally uncoupled from the PSA) is a characteristic stress response of certain phototrophic microorganisms. Two species of green microalgae from the order Chlamydomonadales are the important biotechnological sources of secondary carotenoids: *Dunaliella salina* (formerly *D. bardawil*) [29,30] and *Haematococcus lacustris* (formerly *H. pluvialis*) [31,32]. Accumulation of secondary carotenoids has been also reported in some other microalgal species: *Coelastrella* spp. (including former *Scotiellopsis*) [22,33,34,35], *Bracteacoccus* spp. [33,36], *Chromochloris zofingiensis* (formerly *Chlorella zofingiensis*) [37,38,39], *Sanguina* spp. [15,16], *Deasonia* sp. [40], *Macrochloris* spp. [40], *Tetrahedron minimum* [41], and some strains identified as *Scenedesmus* sp. [42,43], *Muriellopsis* sp. [44]. It is commonly accepted that the carotenogenic response represents an adaptation to stress [45] independently acquired by phylogenetically distant microalgal species during their evolution [46].

Carotenogenic algae are considered as model objects for studies of the response of PSA to various stress factors, especially UV radiation. However, the data on the effect of UV-A on different groups of carotenogenic microalgae are poor. The response of PSA is especially interesting as it is the main target of damage by harmful radiation. The present study aims at elucidating the effects of UV-A on the PSA of three carotenogenic microalgae from different families of Chlorophyta originated from contrasting habitats, namely *H. rubicundus* Allewaert et Vanormelingen (an aeroterrestrial alga), *Bracteacoccus aggregatus* Tereg (a freshwater alga), and *Deasonia* sp. (a soil alga). Toward this end we exposed algal cultures to artificial light with adding of UV-A. Then, water-methanol (polar) and chloroform (non-polar) fractions of the extracts of the microalgae were analyzed by absorbance spectroscopy. It provided the data on changes in the composition of hydrophobic and hydrophilic compounds which absorb radiation, i.e., photosynthetic pigments and photoprotectants. Photosynthetic primary photochemistry also was evaluated by the analysis of chlorophyll fluorescence induction kinetics during the UV-A treatment.

In the Results section we provide the data of visual observations of algal cultures as well as microscopic observations of algal cells during 21 day of UV-A treatment (Section 2.1), describe changes of light absorbing properties and pigment indices based on the analysis of the chloroform extracts (Section 2.2), describe the changes of UV absorption by the water-methanol extracts (Section 2.3), and showed the changes of the parameters of PSII primary photochemistry based on the chlorophyll fluorescence induction kinetics analysis (Section 2.4). Obtained data are discussed and compared with previously published results in the Section 3. Experimental details of the work are explained in the Section 4. Obtained conclusions are summarized in the Conclusion (Section 5).

## 2. Results

### 2.1. Microscopic Observations

**Figure 1:**
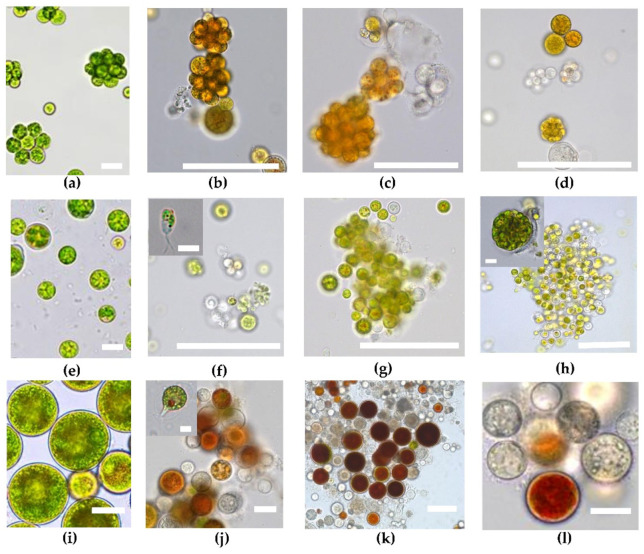
Representative microphotographs of the cells of the strains of carotenogenic microalgae treated by UV-A. (**a**–**d**) *Deasonia* sp. NAMSU 934/2; (**e**–**h**) *Bracteacoccus aggregatus* BM5/15; (**i**–**l**) *Haematococcus rubicundus* BM7/13. (**a**,**e**,**i**) 0 day, (**b**,**f**,**j**) 7 day, (**c**,**g**,**k**) 14 day, (**d**,**h**,**l**) 21 day. Scale bar: 20 μm. Inserts: (**f**,**j**) zoospores, (**h**) autosporangium, scale bar: 2 μm.

The microphotographs from each experiment stage were obtained for all studied strains: *Deasonia* sp. NAMSU 934/2 (Figure 1a–d), *B. aggregatus* BM5/15 (Figure 1e–h) and *H. rubicundus* BM7/13 (Figure 1i–l).

In the beginning of the experiment all cultures were characterized by their typical cell morphology. Thus, vegetative cells of *Deasonia* sp. NAMSU 934/2 were immotile, coccoid, 5–40 μm in diameter (Figure 1a); asexual reproduction via autosporangia was observed. They contained from 4 to 32 autospores. The culture consisted of either single cells or, more frequently, tetrahedral (or occasionally ring-like) colonies. In the culture of *B. aggregatus* BM5/15 small spherical coccoid cells 5–10 μm in diameter persisted (Figure 1e). Occasionally, large cells up to 25 μm diameter also were observed. As a rule, *B. aggregatus* cells formed large clusters. *H. rubicundus* BM7/13 was presented by individual spherical vegetative cells up to 50 μm in diameter (Figure 1i).

The most pronounced feature of the *H. rubicundus* BM7/13 and *B. aggregatus* BM5/15 cultures was the mass production of bi-flagellated zoospores at the 7th d of UV-A treatment (Figure 1f,j). Particularly, zoosporangia with motile zoospores were observed occasionally (Figure 1f). Later, the zoospores disappeared abruptly and were not detected after 14 (Figure 1g,k) or 21 (Figure 1l) days of incubation. At the same time, vegetative cells were smaller than before UV-A treatment. The same was true for *Deasonia* sp. NAMSU 934/2 (Figure 1b–d). UV-A irradiation led to bleaching of the cells in the cultures of *H. rubicundus* BM7/13 and *Deasonia* sp. NAMSU 934/2 (Figure 1 d,l). The bleaching was less pronounced in the culture of *B. aggregatus* BM5/15 characterized by a high degree of cell aggregation (Figure 1f–h). The cells at the core of BM5/15 cell clusters were pigmented, whereas the peripheral cells were bleached and dead (Figure 1h).

No significant changes in cell morphology were observed in the control experiment, when the cells of *H. rubicundus* BM7/13, *Deasonia* sp. NAMSU 934/2 and *B. aggregatus* BM5/15 were cultured under the same conditions, but without UV-A treatment (Supplementary File S1).

### 2.2. Cell Pigment Composition

**Figure 2:**
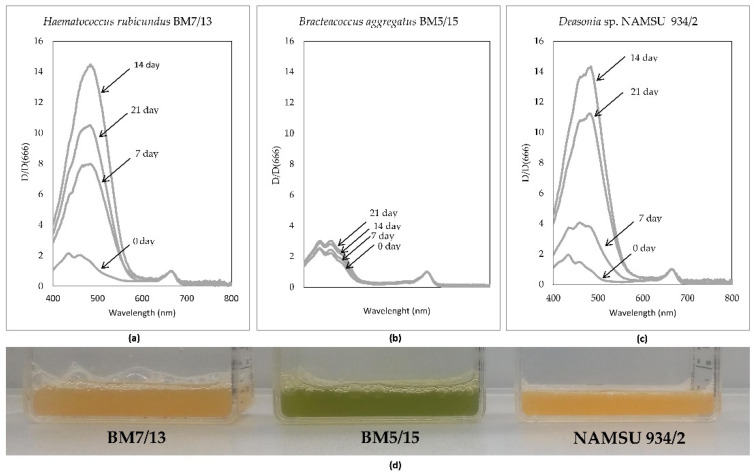
Light absorption by the pigments of the cells of the strains of carotenogenic microalgae treated by UV-A. Absorbance spectra of chloroform extracts of (**a**) *Haematococcus rubicundus* BM7/13, (**b**) *Bracteacoccus aggregatus* BM5/15, (**c**) *Deasonia* sp. NAMSU 934/2 at different days of cultivation. (**d**) Cell suspensions of the carotenogenic microalgae after 14 days of incubation under UV-A.

To evaluate the changes in the pigment composition of the studied strains during UV-A-treatment, their chloroform extracts were studied. absorbance spectra of the extracts were obtained in the visible and near infrared (NIR) range for all three carotenogenic microalgae: *H. rubicundus* BM7/13 (Figure 2a), *B. aggregatus* BM5/15 (Figure 2b) and *Deasonia* sp. NAMSU 934/2 (Figure 2c).

No significant changes in the color of cell suspensions were observed in the color experiment without the treatment by UV-A (Supplementary File S1). Under the UV-A treatment, the pigment composition of *H. rubicundus* BM7/13 and *Deasonia* sp. NAMSU 934/2 changed dramatically (Figure 2d). Visually, it appeared as a pronounced change of color from green to pink or orange (Figure 2d). It was not the case in *B. aggregatus* BM5/15, which retained its green color throughout 21 days of the experiment (Figure 2d). The visual observation was confirmed by the analysis of the absorbance spectra of chloroform cell extracts (Figure 2a–c). UV-A treatment of *H. rubicundus* BM7/13 and *Deasonia* sp. NAMSU 934/2 (Figure 2a,c) cultures led to an increase in the extract absorbance in the blue-green region of the spectrum reflecting the accumulation of secondary ketocarotenoids. Similarly pronounced changes lacked in the spectra of *B. aggregatus* BM5/15 cell extracts (Figure 2b). Based on the spectral features, this culture did not accumulate ketocarotenoids. Statistically significant decrease of chlorophyll content was observed in all cultures during the treatment by UV-A (Supplementary File S2).

**Figure 3:**
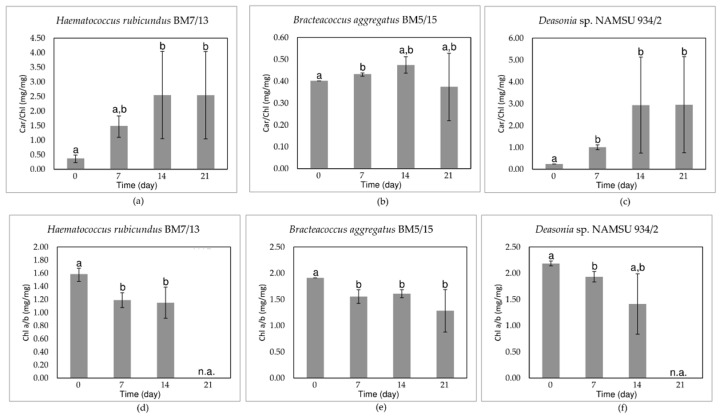
Pigment-based physiological indices of the cells of the strains of carotenogenic microalgae treated by UV-A: (**a**–**c**) the Car/Chl Ratio; (**d**–**f**) The Chl *a*/*b* ratio; (**a**,**d**) *Haematococcus rubicundus* BM7/13; (**b**,**e**) *Bracteacoccus aggregatus* BM5/15; (**c**,**f**) *Deasonia* sp. NAMSU 934/2. Average values and standard deviations are shown. The values from the statistically similar pairs of data for the same microalgal strains are market by same letters.

The calculated Car/Chl ratio served as a proxy to the extent of carotenoid accumulation (Figure 3a–c). The strains *H. rubicundus* BM7/13 and *Deasonia* sp. NAMSU 934/2 were characterized by a sharp rise of this parameter after seven days of UV-A treatment (Figure 3a,c). Then, these cultures were characterized by high dispersion of the parameter (Figure 3a–c). Most likely, it was attributed to the massive cell death and destruction of the pigments. The Car/Chl of the *B. aggregatus* BM5/15 cultures did not change so sharp. However, statistically significant difference was observed between 7th and initial days (Figure 3b).

The UV-A treatment led to a decrease of the ratio of Chl *a* to Chl *b* content (Chl *a*/*b* ratio) in *H. rubicundus* BM7/13 (Figure 3d), *B. aggregatus* BM5/15 (Figure 3e), and *Deasonia* sp. NAMSU 934/2 (Figure 3f). After a decrease of this parameter recorded for the initial seven days of cultivation, it showed no significant change thereafter. At the 21st day of cultivation under UV-A exposure the ration was not detected for *H. rubicundus* BM7/13 (Figure 3d) and *Deasonia* sp. NAMSU 934/2 (Figure 3f) due to significant degradation of Chl *a* and Chl *b* (Supplementary File S2).

### 2.3. The Spectra of Water-Methanol Extracts

**Figure 4:**
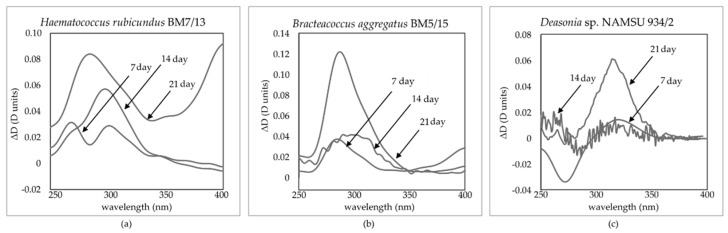
Differential spectra of water-methanol fractions (against 0 day) of the cells of the strains of carotenogenic microalgae treated by UV-A: (**a**) *Haematococcus rubicundus* BM7/13; (**b**) *Bracteacoccus aggregatus* BM5/15; (**c**) *Deasonia* sp. NAMSU 934/2.

**Figure 5:**
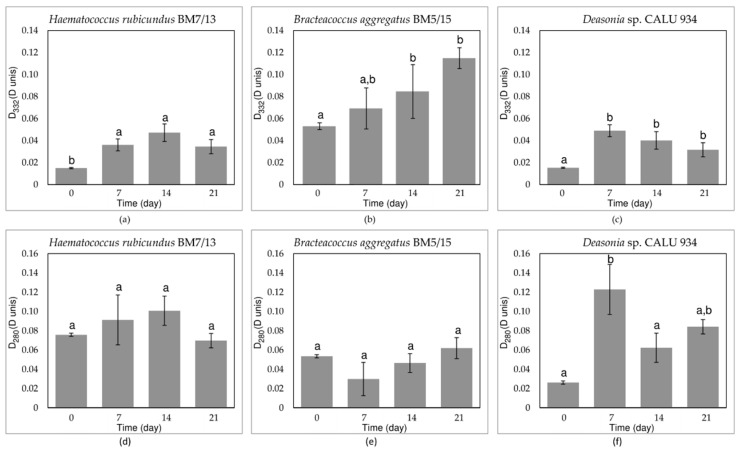
Absorbance of water-methanol fractions of the cells of the strains of carotenogenic microalgae treated by UV-A at (**a**–**c**) 332 nm and (**d**–**f**) 280 nm; (**a**,**d**) *Haematococcus rubicundus* BM7/13; (**b**,**e**) *Bracteacoccus aggregatus* BM5/15; (**c**,**f**) *Deasonia* sp. NAMSU 934/2. Average values and standard deviations are shown. The values from the statistically similar pairs of data for the same microalgal strains are market by same letters.

To evaluate the changes in the pigment composition of the studied strains during UV-A-treatment, their chloroform extracts were studied. absorbance spectra of the extracts were obtained in the visible and near infrared (NIR) range. While in the chloroform extracts pronounced changes was observed in the light absorbance in the visible range, the water-methanol fractions from different days of incubation were different in the UV part of the spectrum. The differential spectra of water-methanol extracts were analyzed to indicate accumulation of non-polar UV-absorbing compounds during UV-A treatment. The spectra were calculated by subtraction of the absorbance spectra of the extract made at the beginning of the experiment (D(λ)^0 day^) from the spectra of extracts of the cells sampled at subsequent days of the experiments (ΔD(λ) = D(λ) − D(λ)^0 day^). The spectra were obtained for all studied strains: *H. rubicundus* BM7/13 (Figure 4a), *B. aggregatus* BM5/15 (Figure 4b) and *Deasonia* sp. NAMSU 934/2 (Figure 5c). The absorbances at 332 nm (Figure 5a–c) and 280 nm (Figure 5d–f) also were monitored for the extracts. In *H. rubicundus* BM7/13 (Figure 4a) and *Deasonia* sp. NAMSU 934/2 (Figure 4c). The UV-A treatment was accompanied by an increase in the absorbance in the range of 270–290 nm attributable to the accumulation of polar, likely aromatic compounds. At the same time, *B. aggregatus* BM5/15 extracts were characterized by increased absorption in the band of 300–350 nm (Figure 4b) likely indicative of MAA accumulation. A less pronounced inflection (“shoulder”) in this spectral range was also detected in the extracts from other two studied algae.

The absorbance at 332 nm, also potentially indicative of MAA presence, increased gradually throughout the experiment in the case of *B. aggregatus* BM5/15 (Figure 5b). In *H. rubicundus* BM5/15 (Figure 5a) extracts it increased after 7 days of culturing and then changed insignificantly. Its sharp rise was also observed in *Deasonia* sp. NAMSU 934/2 (Figure 5c), then the absorbance changing was not statistically significant. In the one-way ANOVA of the data on the absorbance at 280 nm, certain groups of statistically different datasets (corresponded to days of cultivation) were distinguished (*p* < 0.05) only for *Deasonia* sp. NAMSU 934/2. The data on the extracts from *H. rubicundus* BM7/13 (Figure 5d) and *B. aggregatus* BM5/15 cells were statistically homogeneous (Figure 5e). The absorbance at 280 nm increased slightly in *Deasonia* sp. NAMSU 934/2 (Figure 5f) on the 7th day of UV-A treatment. The decrease on the 2nd and 3rd weeks of culturing could be explained by cell death and release of UV-absorbing compounds to the culture medium.

### 2.4. Chlorophyll Fluorescence Induction Curves

**Figure 6:**
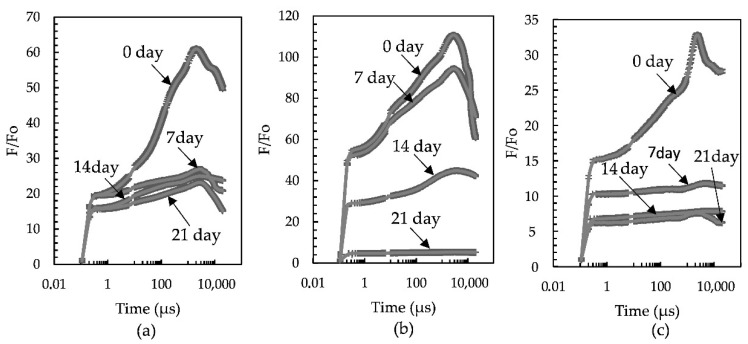
Chlorophyll fluorescence curves (OJIP-curves) of the cells of the strains of carotenogenic microalgae treated by UV-A: (**a**) *Haematococcus rubicundus* BM7/13; (**b**) *Bracteacoccus aggregatus* BM5/15; (**c**) *Deasonia* sp. NAMSU 934/2. Average values and standard deviations are shown.

The Chl fluorescence transient curves (OJIP-curves) were recorded for the studied UV-A-treated carotenogenic microalgae at all stages of the experiment: *H. rubicundus* BM7/13 (Figure 6a), *B. aggregatus* BM5/15 (Figure 6b) and *Deasonia* sp. NAMSU 934/2 (Figure 6c).

The amplitude of the Chl fluorescence transient curves decreased sharply after first seven days of UV-A treatment in the cultures of *H. rubicundus* BM7/13 and *Deasonia* sp. NAMSU 934/2 (Figure 6a,c). This decrease was less pronounced in the *B. aggregatus* BM5/15 culture (Figure 6b). The characteristic shape of the fluorescence transient curve was retained until 14 days of incubation (Figure 6b).

The UV-A treatment led to a decrease of photosynthetic activity measured as the maximal PSII photochemical quantum yield in the dark-acclimated state (Fv/Fm) in all cases. In *H. rubicundus* BM7/13 and *Deasonia* sp. NAMSU 934/2, Fv/Fm decreased sharply after the first seven days of the incubation under UV-A (Table 1) approaching zero by last days of the experiment. In the *B. aggregatus* BM5/15 culture, Fv/Fm was as low as 0.4, i.e., lower than in the other cases (Table 1) but its decrease was less abrupt. The photosynthetic activity was retained in *B. aggregatus* BM5/15 cultures until the last day of the experiment. As in the case of Fv/Fm, *H. rubicundus* BM7/13 and *Deasonia* sp. NAMSU 934/2 were characterized by a sharp decline of the probability of electron transport beyond Q_A_ (Ψ_0_) during UV-A treatment (Table 1). It was most pronounced in the *Deasonia* sp. NAMSU 934/2 reflect likely an effect of UV-A on PSA at the level of electron transport in the PSII RC. At the same time, *B. aggregatus* BM5/15 retained relatively high values of this parameter, because there were statistically different groups of data based on the ANOVA analysis (*p* < 0.05) (Table 1). Thus, UV-A exposure of *B. aggregatus* BM5/15 did not lead to complete inhibition of photosynthesis. The parameter ABS/RC (effective PS II antenna size or its absorbance cross-section) gradually decreased during UV-A treatment in the cultures of *H. rubicundus* BM7/13 and *B. aggregatus* BM5/15. In the case of *B. aggregatus* BM5/15 this decrease was less pronounced. By contrast, a sharp rise of ABS/RC was observed in *Deasonia* sp. NAMSU 934/2 on the 7th day but the values of this parameter varied significantly. On the 21st day, its value decreased sharply. In *H. rubicundus* BM7/13 and *Deasonia* sp. NAMSU 934/2 cultures, the UV-A irradiation led to a decrease in the Stern-Volmer non-photochemical quenching parameter, NPQ (Table 1). At the same time, this parameter increased in *B. aggregatus* BM5/15 (Table 1). Thus, UV-A treatment could activate non-photochemical quenching of the excited Chl states (NPQ) in *B. aggregatus* BM5/15, whereas in *H. rubicundus* BM7/13 and *Deasonia* sp. NAMSU 934/2 NPQ was downregulated.

No significant difference was observed in the values of main parameters of Chl fluoresce curves in the control experiment (without UV-A treatment) (Supplementary File S1).

## 3. Discussion

Overall, UV-A is less harmful for microalgae than UV-B [47,48,49]. Different groups of phototrophs are characterized by different responses of their PSA to UV [50]. To further understand the diversity and specificity of the UV-A effect on the algal PSA, it was studied in the three ecologically and phylogenetically distant strains of carotenogenic microalgae.

The data on UV-A effect on microalgae are scarce. UV-A + UV-B treatment of the cyanobacterium *Aphanizomenon flos-aquae*, the green algae *Selenastrum capricornutum* (Chlorophyceae) [51], *Micromonas polaris* (Mamiellophyceae) have been published [13]. Physiological responses of UV-A were described in *Dunaliella salina* (Chlorophyceae) [23,49,52,53,54,55,56], *Phaeocystis globosa* (Prymnesiophyceae) [53], *Coelastrella rubescens* (Chlorophyceae) [22], *Nannochloropsis oceanica* (Eustigmatophyceae) [11], *Chloromonas krienitzii* [15] and some strains of *Chlorella* spp. (Trebouxiophyceae) [48]. Here, we report on a comparative study of the UV-A response in three strains of ketocarotenoid-producing microalgae. All these microalgae are characterized by a high stress resilience, but they differed in their patterns of UV-A stress response.

In two of the studied strains, an increase in Car/Chl ratio was observed. In case of primary carotenoids, its value is strictly defined by conserved stoichiometric relationships between the carotenoids and Chl in PSA pigment-protein complexes [54,55,57]. UV-A-induced rise of Car/Chl has been reported in other algae [22,24,49,52,56]. Its moderate increase can be due to the synthesis of additional primary carotenoids, such as those participation in xanthophyll cycles. Expectedly, a pronounced accumulation of secondary carotenoids was recorded in the two of three studied microalgal strains (*H. rubicundus* BM7/13 and *Deasonia* sp. NAMSU 934/2).

Another carotenogenic microalga, *D. salina*, under a moderate UV-A exposure showed an increase in the content of major cell carotenoids, especially lutein and β-carotene, as well as in the pigments of violaxanthin cycle [49,52,56]. At the same time, high doses of UV-A promoted only the accumulation of β-carotene as the secondary carotenoid [49]. Similar effect was also shown for *C. rubescens* [22] and *Ch. krienitzii* [15]. At the ultrastructural level, UV-A treatment in the carotenogenic algae *C. rubescens* and *Ch. krienitzii* was accompanied by an expansion of the hydrophobic (sub)compartments of the cell including cytoplasmic oil bodies and plastoglobuli harboring the secondary carotenoids [15,22]. Accumulated secondary carotenoids absorb excessive UV-A and shield PSA. Thus, *B. aggregatus* BM5/15 demonstrated an effect typical of moderate UV-A treatment, the same doses of UV-A exerted a deteriorative effect on *H. rubicundus* BM7/13 and *Deasonia* sp. NAMSU 934/2.

In phototrophs, the actual photosynthetic performance is determined by the balance of UV-A and visible light irradiation. When the visible light is limiting, UV-A is favorable for photosynthesis, but under over-saturating visible light intensities UV-A exposure leads to photoinhibition [5]. UV-A has a bi-phasic effect on PSA [1,2,5,9,10,11]. It has been shown previously especially for microalgae. In *N. oceanica* moderate UV-A doses promote culture growth, as well as upregulating overall metabolic activity, estimated as O_2_ photosynthetic emission, and nitrate assimilation [11]. The moderate doses also increase the rate of the O_2_ production in *D. salina* [49,52]. In *P. globosa* moderate increase of the UV-A dose leads to an increase of photosynthetic activity and decrease of NPQ [53]. At the same time, in the same species of microalgae high UV-A downs out photosynthesis [11,23,49].

In all studied cases, the UV-A treatment led to a decrease of the potential photochemical efficiency of PSII (measured as Fv/Fm), this effect was also documented in the present work and in *C. rubescens* under the same experimental conditions [22]. Mechanistically, the harmful effect of UV-A on PSA is thought to be implemented mainly by PSII RC damage [9,13,52,56,58,59]. The Fv/Fm parameters provides only information about the potential maximal photochemical efficiency of PSII, which is often not sufficient; such cases require additional parameters such as Ψ_0_ to be considered. Thus, a pronounced difference of Ψ_0_ was observed in the studied strains. While *H. rubicundus* BM7/13 and *Deasonia* sp. NAMSU 934/2 demonstrated a sharp decrease of Ψ_0_, *B. aggregatus* BM5/15 retained moderately high values of this parameter. Ψ_0_ reflects the efficiency of the electron transport between Q_A_ and Q_B_ in PSII [60]. Indeed, UV-A predominantly affects the OEC, Q_A_ and Q_B_ binding sites in PSII [9,10,14]. Accordingly, the data obtained in this study reinforce the current view of the UV effect on PSA, although in *B. aggregatus* BM5/15 this effect was less pronounced.

In all studied cultures, the UV-A irradiation promoted the decrease in Chl *a*/*b*. Since Chl *b* is contained exclusively in the light-harvesting antenna (LHC), this effect suggests a relative increase of the antenna size. However, the effective PSII antenna size (ABS/RC) calculated from the Chl fluorescence transient curves decreased in all cases. Most likely, the algal cells responded to the UV-A stress by reducing the amount of the absorbed PAR and hence by lowering the excitation pressure on PSII and subsequent ROS formation. At the same time, Chl *a*/*b* decrease could reflect increasing could be explained by the decreasing of the PSI antennae size. PSI upregulation might be important to weaken over-reduction of the plastid electron transport chain. Ivanova et al. [10] have shown the decrease of antenna size facilitates the protection of PSA against UV-A damage in a legume *Pisum sativum*. Uncoupling of LHCII complexes and their oligomerization is considered as an additional UV-protective mechanism [10].

Non-photochemical quenching of the excited Chl states is an omnipresent photoprotective mechanism in photosynthetic organisms [61]. It redirects the excessively absorbed light energy from destructive photochemical reactions to safe thermal dissipation. Under our experimental conditions, *H. rubicundus* BM7/13 and *Deasonia* sp. NAMSU 934/2 were not characterized by the increase of the Stern-Volmer NPQ parameter under the UV-A exposure. This finding suggests a downregulation and/or inhibition of non-photochemical quenching. By contrast, in *B. aggregatus* BM5/15, NPQ increased suggesting upregulation of the thermal dissipation. Under the UV-A treatment, *P. globosa* [53], *N. oceanica*, and *C. rubescens* also decreased their NPQ [22,49,53]. This effect was observed despite of the expansion of the violaxanthin cycle pigment pool responsible, at least partially, for NPQ development [56,59]. However, in chlorophytes the xanthophyll cycle seems to be less important for the NPQ engagement than in higher plants [62,63]. In view of this, the response of *B. aggregatus* BM5/15 to UV-A can be regarded as unusual. It could be explained by the upregulation of NPQ to protect its highly functional PSA, whereas two other strains did not need it since their photosynthetic activity declined dramatically.

Under the UV-A treatment, an increase in the absorbance at 332 nm was observed in all studied strains. This response was most pronounced in *B. aggregatus* BM5/15. Most likely, absorbance at this wavelength together with shape of the spectra could be attributed to the accumulation of MAA. These compounds, widely distributed among microalgae, are well-known as efficient screening agents [4,5,6,18,19,20]. Their protective role, especially against UV, has been shown in vivo [4,18,64]. As a rule, MAA synthesis is UV-inducible [19,20,22]. It is in line with the data obtained in current work.

Although mechanisms of regulation of MAAs synthesis in algae are poorly understood [64], it mediated most likely by the cryptochromes absorbing UV-A [8]. One can speculate that the higher viability and photosynthetic activity of *B. aggregatus* BM5/15 was attributed, inter alia, to a higher MAA level. Nevertheless, it might only be a side effect because the cultures were irradiated by UV-A in the range of 380–415 nm (see Materials and Methods), whereas the absorption band of putative MAA is located in the range of 300–350 nm. However, MAA also have antioxidant effect [4,18] so they could contribute to ROS detoxication. Furthermore, a pronounced increase of the culture extract absorption was observed at 280 nm in *Deasonia* sp. NAMSU 924/2. It could reflect accumulation of simple phenolic compounds such as in some other chlorophytes [65].

Microscopic observations could reveal the relationship between UV radiation and its effect on the microbial physiology and structure. The most spectacular feature under our experimental condition in the *H. rubicundus* and *B. aggregatus* cultures was the mass zoospore production observed on the seventh day of UV-A exposure. Production of motile cells in the carotenogenic *H. lacustris* can be induced by a sharp change of cultivation conditions [66]. Light intensity and spectral quality play a significant role in both sporogenesis and gametogenesis in green algae. Short-wavelength (blue) PAR radiation is favorable for massive formation of flagellated cells, whereas long-wavelength (red) PAR blocks it [26]. In the macrophyte *Bryopsis plumosa* (Ulvophyceae), a positive effect of UV-A on motile stages production has been reported [27]. Thus, zoospore production in the studied alga is another typical response UV-A. From the practical standpoint, UV-A irradiations could be used for the induction and study of motile stages in the cultures of carotenogenic algae.

Another important conclusion from obtained microscopic data is the relationship between UV-A response of cells and their ability to form aggregates. *B. aggregatus* BM5/15 formed large cell clusters by the end of the experiment, moreover autospores in sporangia stayed together under the mother envelope. The external cells of these clusters were bleached or intensely red-colored due to carotenoid accumulation, whereas internal cells remained green. It is likely that the external cells were exposed to high doses of UV-A blocked it by either their photosynthetic pigments or by secondary carotenoids or by UV-absorbing compounds in their walls, such as rigid sporopollenin or algaenan layer [15,22]. Even if damaged or killed by the UV-A exposure, they would continue to shield the internal cells of the cluster against the radiation. Thus, formation of aggregates and autosporangia could be considered as a mechanism for protection against the harmful impact of UV-A. Such protection by self-shielding has been proposed previously for microalgae of biological soil crust and biofilms [3,19].

Notably, the effect of radiation strongly depends on the size of an organism. Larger organisms receive a lower dose than smaller ones under the same radiation flux. Therefore, unicellular algae with larger cell size are more resistant to UV-A and UV-B than those with a smaller size [5]. Accordingly, the colonies of *B. aggregatus* BM5/15 could mimic the elevated UV-A radiation resilience of larger organisms. This ability might be critical to retain the functional PSA and increased viability. By contrast, *H. rubicundus* BM7/13 cultures lacked massive cell aggregates so this might be the explanation of its low viability and inhibited photosynthesis. A high variation of the parameters recorded in the experiment might also reflect a high functional heterogeneity of the cells in the UV-treated cultures. Further research is required to deeper understand the relationships between the species-specific and general patterns of the UV-A response in the photosynthetic microorganisms, especially carotenogenic microalgae, and their cultivation condition.

## 4. Materials and Methods

### 4.1. Strains and Culture Conditions

The following strains of green carotenogenic microalgae were studied: *Haematococcus rubicundus* BM7/13, *Bracteacoccus aggregatus* BM5/15 (IPPAS C-2045), and *Deasonia* sp. NAMSU 934/2. They were isolated and identified previously [33,40]. The cells were taken from the stationary growth phase, because at this growth stage the cultures are characterized by relatively low division rate, and, thus, more resistant to UV radiation [67]. Initial optical density of the cultures was 0.4 as was measured at 660 nm in standard quartz civets in an Agilent Cary 300 spectrophotometer (Agilent, Santa Clara, CA, USA) with an integrative sphere (150 mm diameter) of the same manufacturer. They were cultured autotrophically on the mineral medium BG-11 [68] under continuous illumination by the cold-white LED COB-X544-8mm 24V White6000 (Arlight, Moscow, Russia) (60 μmol/m^2^/s) at 25 °C in the 250 mL T-75 TC-treated cell culture flasks (Eppendorf, Hamburg, Germany) for three weeks. Photon flux density of the visible light was controlled at the level of cell suspensions by a LI-COR LI-250A quantum meter (LI-COR Inc., Lincoln, NE, USA). UV-A treatment was performed by LED 5M 033220 (Arlight, Moscow, Russia) in the spectral range of 380–415 nm with the power of 2.9 W/m^2^. Emission spectrum of the radiation is given in supplementary (Supplementary File S3). All experiments were carried out in three replicates, the measurements were carried out in three replicates. As a control experiment the cultures of microalgae also were grown under the same conditions listed above, but without UV-A treatment (Supplementary File S1).

### 4.2. Microscopy

The cultures were monitored by bright-field microscopy on a Leica DM2500 microscope (Leica Microsystems, Wetzlar, Germany) with the attached Leica DFC 700T camera.

### 4.3. Absorbance Spectroscopy

Extraction of hydrophilic and hydrophobic compounds and registration of their absorbance spectra were carried out as was previously described by Zaytseva et al. [22]. The spectra were registered on an Agilent Cary 300 in the UV-visible-NIR range. Differential absorbance spectra (ΔD(λ)) in the UV range (250–400 nm) were presented as ΔD(λ) = D(λ) − D(λ)^0 day^, where D(λ)^0 day^ is the optical density at the wavelength λ in the control (initial day of cultivation). The standard spectral indices (Car/Chl and Chl *a*/*b*) were calculated as was previously described [36].

### 4.4. Chlorophyll Fluorescence Analysis

The Chl fluorescence induction curves were registered by the FluorPen FP100s PAM-fluorometer (Photon System Instruments, Drásov, Czech Republic) as described previously [62] using the protocols for Chl fluorescence transient (OJIP curve) and NPQ induction.

For the OJIP curves the maximal photochemical quantum yield of PS II (Fv/Fm) and the probability of electron transport beyond Q_A_ (Ψ_0_), PS II absorbance cross section, or PS II effective size (ABS/RC) [60,69], were calculated as,

(1) $$\mathrm{Fv}/\mathrm{Fm}=\frac{\mathrm{Fm}-\mathrm{Fo}}{\mathrm{Fm}},$$

where Fm and Fo are maximal and minimal Chl fluorescence intensity, respectively, registered in the dark-acclimated state;

(2) $$\Psi_{0}=1-\frac{F_{J}-\mathrm{Fo}}{\mathrm{Fm}-\mathrm{Fo}},$$

where F_J_ is the Chl fluorescence intensity in the point J (at 2021 μs);

(3) $$\mathrm{ABS}/\mathrm{RC}={\left(\frac{\mathrm{dF}}{\mathrm{dt}}\right)}_{t=0\;\mu s}\times \frac{\mathrm{Fm}}{\left(\mathrm{Fm}-\mathrm{Fo}\right)\times \left(F_{J}-\mathrm{Fo}\right)}\approx \frac{\mathrm{Fm}\times \left(F\left(301\;\mu s\right)-F\left(21\;\mu s\right)\right)}{280\;\mu s\times \left(\mathrm{Fm}-\mathrm{Fo}\right)\times \left(F_{J}-\mathrm{Fo}\right)}.$$

The Stern-Volmer non-photochemical quenching parameter (NPQ) was calculated from the stationary Chl fluorescence curves registered according to the NPQ induction protocol [62] as,

(4) $$\mathrm{NPQ}=\frac{\mathrm{Fm}-{\mathrm{Fm}}^{\prime }}{{\mathrm{Fm}}^{\prime }},$$

where Fm′ is the maximal Chl fluorescence intensity in the light-acclimated state [69]. Minimal values of Fm′ were taken to acquire maximal NPQ for the analysis.

### 4.5. Statistical Treatment

All measurements were performed in total of six replicates (three biological replicates with two analytical replicates in each biological one). The data on each estimated parameter were divided into three independent datasets corresponded to studied strains of microalgae. For analysis of the data of absorbance spectroscopy the standard one-way ANOVA (*p* = 0.95) was applied for the datasets of each parameter for each algal strain. After confirming the hypothesis of differences in certain groups of data, the pairs of samples corresponded to the days of cultivation, were compared by the post-hoc Tukey test. The Kolmogorov-Smirnov test was used to determine normality distribution.

Since the data of chlorophyll fluorescence analysis are not approximated by the normal distribution [70], the non-parametric Kruskal-Wallis H test was applied for the datasets (*p* = 0.99). The standard Mann-Whitney U test commonly used for this type of data [71,72] than was carried out to compare pair of samples.

The analysis was carried out in STATISTICA v. 13.3 (StatSoft, Dell, Round Rock, TX, USA).

## 5. Conclusions

We presented the first work on the effect of UV-A on three phylogenetically distant green ketocarotenoid-producing microalgae, namely *Haematococcus*, *Deasonia*, and *Bracteacoccus*. The genera *Deasonia* and *Bracteacoccus* are poorly studied in terms of their stress physiology.

There were some common features of response to UV-A of three studied strains of green carotenogenic microalgae. (1) In all cases the changes involved PSII primary photochemistry: the decrease of Fv/Fm and $\Psi_{0}$ (reflecting the transfer from Q_A_ to Q_B_), PSII antennae size also decreased. (2) The Chl *a*/*b* ratio decreased, most likely, due to the enlarging og the PSI antennae size. However, this hypothesis should be further tested. (3) All strains accumulated UV-absorbing polar compounds, most likely phenolics and MAAs. The increase of the absorbance at 332 nm (corresponding to MAAs) was most pronounced in *B. aggregatus* BM5/15. At the same time, the strains were different in terms of their response to UV-A. (4) NPQ upregulation was observed only in *B. aggregatus* BM5/15, whereas in other cases its level was low. (5) In all cases carotenoid content increased, but in *H. rubicundus* BM7/13 and *Deasonia* sp. NAMSU 934/2 they were secondary carotenoids, whereas in *B. aggregatus* BM5/15 formed primary ones. In the last cases they could contribute to the NPQ build up. (6) Most pronounced difference was observed in microscopical features of the cultures. *H. rubicundus* BM7/13 and *Deasonia* sp. NAMSU 934/2 were represented mostly by solitaire cells or small cell clusters, which lacked their green color. At the same time, *B. aggregatus* BM5/15 formed aggregates from green cells. Formation of such aggregates could be considered as an additional UV-protecting mechanism. (7). Finally, the strains differed by their viability. *B. aggregatus* BM5/15 was most resistant to UV-A, whereas massive cell death was observed in two other cultures.

## Figures and Tables

**Table 1:** The parameters of the chlorophyll fluorescence induction curves of three strains of carotenogenic microalgae, *Haematococcus rubicundus* BM7/13, *Bracteaoccus aggregatus* BM5/5 and *Deasonia* sp. NAMSU 934/2, treated by UV-A. Median values and ranges between 1st and 3rd quartiles from three biological and three analytical replicates are shown. The values from the statistically similar pairs of data for the same microalgal strains are market by same letters.

Time	Fv/Fm	Ψ_0_	ABS/RC, μs^−1^	NPQ
*Haematococcus rubicundus* BM7/13				
0 d	0.58 (0.58–0.58) ^a^	0.53 (0.53–0.53) ^a^	1.36 (1.36–1.36) ^a^	0.17 (.17–0.17) ^a^
7 d	0.24 (0.13–0.33) ^b^	0.24 (0.22–0.27) ^b^	0.50 (0.34–0.54) ^b^	0.11 (0.08–0.15) ^a,b^
14 d	0.33 (0.08–0.41) ^b^	0.20 (0.20–0.21) ^b^	0.31 (0.23–0.38) ^b^	0.07 (0.07–0.07) ^b^
21 d	0.09 (0.05–0.10) ^c^	0.18 (0.14–0.20) ^b^	0.34 (0.19–0.40) ^b^	0.10 (0.08–0.12) ^a,b^
*Bracteacoccus aggregatus* BM5/15				
0 d	0.41 (0.41–0.41) ^a^	0.27 (0.27–0.27) ^a^	0.86 (0.86–0.86) ^a^	0.07 (0.07–0.07) ^a^
7 d	0.39 (0.35–0.42) ^a^	0.23 (0.22–0.24) ^a^	0.57 (0.19–0.68) ^b^	0.20 (0.02–0.40) ^b^
14 d	0.25 (0.17–0.30) ^b^	0.25 (0.22–0.28) ^a^	0.75 (0.54–0.81) ^a,b^	0.42 (0.38–0.82) ^b,c^
21 d	0.29 (0.19–0.35) ^a,b^	0.31 (0.29–0.34) ^a^	0.55 (0.42–0.60) ^b^	0.38 (0.28–0.44) ^b,c^
*Deasonia* sp. NAMSU 934/2				
0 d	0.70 (0.70–0.70) ^a^	0.44 (0.44–0.44) ^a^	0.54 (0.54–0.54) ^a^	0.10 (0.10–0.10) ^a^
7 d	0.12 (0.09–0.15) ^b^	0.10 (0.08–0.16) ^b^	1.38 (1.22–1.40) ^b^	0.13 (0.10–0.15) ^a^
14 d	0.08 (0.08–0.09) ^b^	0.11 (0.05–0.13) ^b^	0.86 (0.42–0.91) ^a,b^	0.03 (0.02–0.03) ^b^
21 d	0.06 (0.00–0.06) ^b^	0.02 (0.01–0.02) ^c^	0.18 (0.14–0.24) ^d^	0.05 (0.00–0.08) ^a,b^

Fv/Fm—maximal photochemical quantum yield of PS II in the dark-acclimated state; Ψ_0_—probability of electron transport beyond the primary PS II quinone acceptor (Q_A_), ABS/RC—effective PS II antenna size; NPQ—Stern-Volmer non-photochemical quenching parameter.

## Data Availability

Not applicable.

## Supplementary Materials

The following supporting information can be downloaded at: https://www.mdpi.com/article/10.3390/plants11111431/s1, Supplementary File S1: Characterization of the cultures of the carotenogenic microalgae *Haematococcus rubicundus* BM7/13, *Bracteacoccus aggregatus* BM5/15, and *Deasonia* sp. NAMSU 934/2 without UV-A treatment; Supplementary File S2: Pigment content in the cells of three strains of carotenogenic microalgae, *Haematococcus rubicundus* BM7/13, *Bracteacoccus aggregatus* BM5/15, and *Deasonia* sp. NAMSU 934/2 during UV-A treatment; Supplementary File S3: Emission spectrum of the light emitting diodes used in the work.
