# Supplementary material for: Differential Responses to UV-A Stress Recorded in Carotenogenic Microalgae Haematococcus rubicundus, Bracteacoccus aggregatus, and Deasonia sp
Source: Plants (Basel). 2022 May 27;11(11):1431. doi: 10.3390/plants11111431 (PMC9183108; doi:10.3390/plants11111431)
Supplement: Supplementary file 1 [file plants-11-01431-s001.zip › suppl file/Supplementary file S1.pdf]

Supplementary S1. Characterization of the cultures of the carotenogenic microalgae *Haematococcus rubicundus* BM7/13, *Bracteacoccus aggregatus* BM5/15, and *Deasonia* sp. NAMSU 934/2 without UV-A treatment

The following strains of green carotenogenic microalgae were studied: *Haematococcus rubicundus* BM7/13, *Bracteacoccus aggregatus* BM5/15 (IPPAS C-2045), and *Deasonia* sp. NAMSU 934/2. They were isolated and identified previously [1-3].

The conditions were the same as in the experiment, but without UV-A treatment. The cells were taken from the stationary growth phase, because at this growth stage the cultures are characterized by relatively low division rate, and, thus, more resistant to UV radiation [4]. Initial optical density of the cultures was 0.4 as was measured at 660 nm in standard quartz civets in an Agilent Cary 300 spectrophotometer (Agilent, Santa Clara, CA, USA) with an integrative sphere (150 mm diameter) of the same manufacturer. They were cultured autotrophically on the mineral medium BG-11 [5] under continuous illumination by the cold-white LED COB-X544-8mm 24V White6000 (Arlight, Moscow, Russia) (60 μmol/m^2/s) at 25 °C in the 250 mL T-75 TC-treated cell culture flasks (Eppendorf, Hamburg, Germany) for three weeks (Figure S1-1a). Photon flux density of the visible light was controlled at the level of cell suspensions by a LI-COR LI-250A quantum meter (LI-COR Inc., Lincoln, NE, USA).

There were no pronounced changes in the colors of the suspensions of microalgal cells as in the case of the UV-A treatment (Figure S1-1). The cultures demonstrated similar green color at the 7th (Figure S1-1b), 14th (Figure S1-1c) and 21st day (Figure S1-1d) of culturing as at the initial point of the experiment (Figure S1-1a), which is typical for non-stressed cells [1-3].

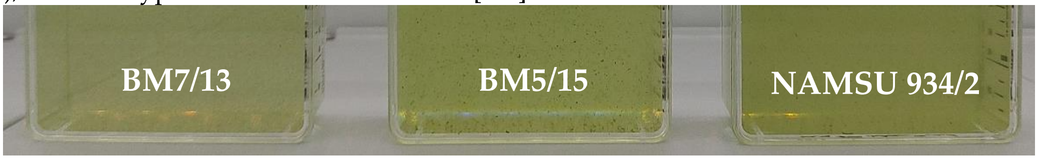

(a)

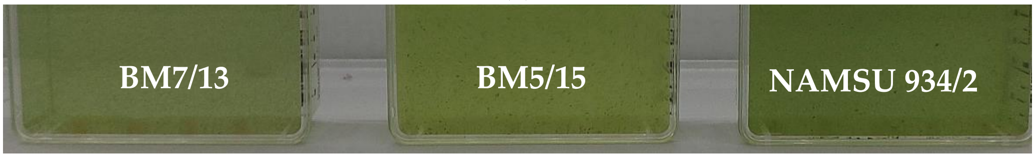

(b)

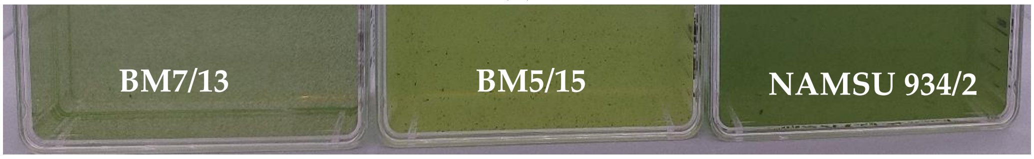

(c)

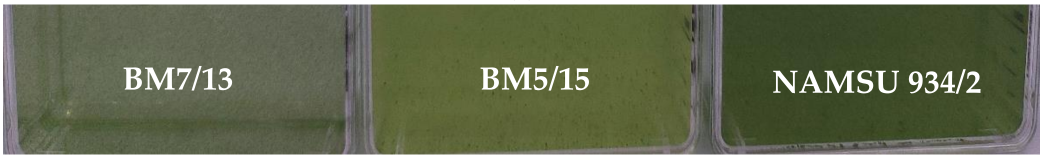

(d)

Figure S1-1. Cell suspensions of the carotenogenic microalgae (**a**) at the initial point, (**b**) after 7th, (**c**) 14th, and 21st (**d**) day of cultivation in the control experiment (without UV-A treatment).

The control cultures were also monitored by bright-field microscopy on a Leica DM2500 microscope (Leica Microsystems, Wetzlar, Germany) with the attached Leica DFC 700T camera (Figure S1-2).

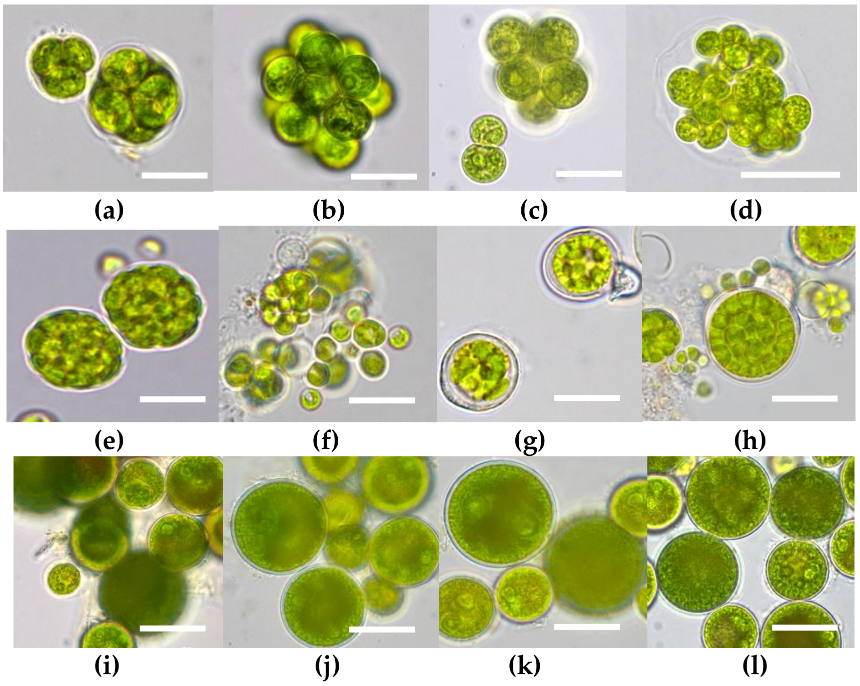

Figure S1-2. Representative microphotographs of the cells of the strains of carotenogenic microalgae in the control experiment (without UV-A treatment). (**a,b,c,d**) *Deasonia* sp. NAMSU 934/2; (**e,f,g,h**) *Bracteacoccus aggregatus* BM5/15; (**i,j,k,l**) *Haematococcus rubicundus* BM7/13. (**a,e,i**) 0 day, (**b,f,j**) 7 day, (**c,g,k**) 14 day, (**d,h,l**) 21 day. Scale bar: 20 μm.

The microphotographs were obtained for all studied strains: *Deasonia* sp. NAMSU 934/2 (Figure S1-2 a,b,c,d), *B. aggregatus* BM5/15 (Figure S1-2 e,f,g,h) and *H. rubicundus* BM7/13 (Figure S1-2 i,j,k,l) for 0 day (Figure S1-2 a,e,i), 7 day (Figure S1-2 b,f,j), 14 day (Figure S1-2 c,g,k), 21 day (Figure S1-2 d,h,l). No significant changes in cell morphology were observed in the control experiment, when the cells of *H. rubicundus* BM7/13, *Deasonia* sp. NAMSU 934/2 and *B. aggregatus* BM5/15 were cultured under the same conditions, but without UV-A treatment.

The Chl fluorescence induction curves were registered by the Fluorpen FP100s PAM-fluorometer (Photon System Instruments, Drásov, Czech Republic) as described previously [3] using the protocols for Chl fluorescence transient (OJIP curve).

The maximal photochemical quantum yield of PS II (Fv/Fm) and the probability of electron transport beyond Q_A (Ψ_0), PS II absorbance cross section, or PS II effective size (ABS/RC) [6,7], were calculated as

$$\mathrm{Fv/Fm} = \frac{\mathrm{Fm-Fo}}{\mathrm{Fm}}, \quad (1)$$

where Fm and Fo are maximal and minimal Chl fluorescence intensity, respectively, registered in the dark-acclimated state;

$$\Psi_0 = 1 - \frac{\mathrm{F_J-Fo}}{\mathrm{Fm-Fo}}, \quad (2)$$

where F_J is the Chl fluorescence intensity in the point J (at 2021 μs).

No significant difference was observed in the values of Fv/Fm and Ψ_0 (Table S1). Both parameters retained high values for all three strains of microalgae.

Table S1. The parameters of the chlorophyll fluorescence induction curves of three strains of carotenogenic microalgae, *Haematococcus rubicundus* BM7/13, *Bracteaoccus aggregatus* BM5/5 and *Deasonia* sp. NAMSU 934/2 under the control conditions (without UV-A treatment).

**Time**	**Fv/Fm**	**Ψ_0**
Haematococcus rubicundus BM7/13		
0 d	0.58	0.39
7 d	0.66	0.38
14 d	0.64	0.41
21 d	0.61	0.42
Bracteacoccus aggregatus BM5/15		
0 d	0.65	0.56
7 d	0.62	0.58
14 d	0.63	0.55
21 d	0.61	0.56
Deasonia sp. NAMSU 934/2		
0 d	0.62	0.41
7 d	0.62	0.43
14 d	0.67	0.45
21 d	0.64	0.44

* Fv/Fm – maximal photochemical quantum yield of PS II in the dark-acclimated state; Ψ_0 – probability of electron transport beyond the primary PS II quinone acceptor (Q_A),

References

1. Chekanov, K.; Fedorenko, T.; Kublanovskaya, A.; Litvinov, D.; Lobakova, E. Diversity of carotenogenic microalgae in the White Sea polar region. *FEMS Microbiol. Ecol.* **2020**, *96*(1), fiz183.
2. Shibzukhova, K.A.; Gavrilova, O.V.; Chivkunova, O.B.; Sidorov, R.A. Solovchenko, A.E.; Lobakova, E.S. Estimation of biotechnological potential and clarification of taxonomic status of *Parietochloris* genus microalgae (Trebouxiophyceae) from the NAMSU collection. *Mosc. Univ. Biol. Sci.* **2017**, *72*(3), 137-141.

3. Chekanov, K.; Lukyanov, A.; Boussiba, S.; Aflalo, C.; Solovchenko, A. Modulation of photosynthetic activity and photoprotection in *Haematococcus pluvialis* cells during their conversion into haematocysts and back. *Photosynth. Res.* 2016, *128*(3), 313-323.
4. Woodwell, G.M.; Whittaker, R.H. Effects of chronic gamma irradiation on plant communities. *Q. Rev. Biol.* **1968**, *43*(1), 42-55.
5. Stanier, R.Y.; Kunisawa, R.; Mandel, M.C.B.G.; Cohen-Bazire, G. Purification and properties of unicellular blue-green algae (order *Chroococcales*). *Bacteriol. Rev.* **1971**, 35, 171–205.
6. Strasser, R.J.; Tsimilli-Michael, M.; Srivastava, A. Analysis of the chlorophyll a fluorescence transient. In *Chlorophyll a fluorescence*; Papageorgiou, G.C., Govindjee, Eds.;. Springer: Dordrecht, Germany, 2007, pp. 321-362.
7. Maxwell, K.; Johnson, G.N. Chlorophyll fluorescence—a practical guide. *J. Exp. Bot.* **2000**, *51*(345), 659-668.

Table S2. The contents of main groups of pigments in the cells of three strains of carotenogenic microalgae, *Haematococcus rubicundus* BM7/13, *Bracteacoccus aggregatus* BM5/15, and *Deasonia* sp. NAMSU 934/2 during UV-A treatment. Average values and standard deviations are shown. The data from the same statistical groups for the same microalgal strains are market by same letters.

Time	Chlorophyll *a* (mg/L)	Chlorophyll *b* (mg/L)	Carotenoids (mg/L)
Haematococcus rubicundus BM7/13			
0 d	2.81±0.23[a]	1.78±0.12[a]	1.74±0.38[a]
7 d	0.20±0.04[b]	0.17±0.02[b]	0.57±0.12[a]
14 d	0.21±0.04[b]	0.20±0.09[b]	0.92±0.38[a]
21 d	0.10±0.02[c]	0.09±0.00[b]	0.6±0.20[a]
Bracteacoccus aggregatus BM5/15			
0 d	0.88±0.00[a]	0.46±0.00[a]	0.54±0.00[a]
7 d	0.59±0.12[b]	0.38±0.010[a]	0.41±0.06[a]
14 d	0.89±0.33[a,b]	0.55±0.19[a]	0.68±0.25[a]
21 d	1.02±0.09[a,b]	0.70±0.52[a,b]	0.66±0.39[a,b]
Deasonia sp. NAMSU 934/2			
0 d	1.32±0.28 [a]	0.60±0.14 [a]	0.46±0.10 [a]
7 d	0.89±0.12 [a]	0.46±0.06 [a]	1.36±0.07 [a]
14 d	0.46±0.51 [a]	0.53±0.76 [a]	1.10±0.02 [a]
21 d	0.24±0.01 [a]	0.14±0.01 [a]	1.15±0.10 [a]

References

8. Zaytseva, A.; Chekanov, K.; Zaytsev, P.; Bakhareva, D.; Gorelova, O.; Kochkin, D.; Lobakova, E. Sunscreen effect exerted by secondary carotenoids and mycosporine-like amino acids in the aeroterrestrial chlorophyte *Coelastrella rubescens* under high light and UV-A irradiation. *Plants* **2021**, *10*(12), 2601.

9. Chekanov, K.; Litvinov, D.; Fedorenko, T.; Chivkunova, O.; Lobakova, E. Combined production of astaxanthin and β-carotene in a new strain of the microalga *Bracteacoccus aggregatus* BM5/15 (IPPAS C-2045) cultivated in photobioreactor. *Biology* **2021**, *10*(7), 643.
