# Supplementary material for: Differential Responses to UV-A Stress Recorded in Carotenogenic Microalgae Haematococcus rubicundus, Bracteacoccus aggregatus, and Deasonia sp
Source: Plants (Basel). 2022 May 27;11(11):1431. doi: 10.3390/plants11111431 (PMC9183108; doi:10.3390/plants11111431)
Supplement: Supplementary file 1 [file plants-11-01431-s001.zip › suppl file/Supplementary file S2.pdf]

Supplementary S2. Pigment content in the cells of three strains of carotenogenic microalgae, *Haematococcus rubicundus* BM7/13, *Bracteacoccus aggregatus* BM5/15, and *Deasonia* sp. NAMSU 934/2 during UV-A treatment

Extraction of hydrophilic and hydrophobic compounds and registration of their absorbance spectra were done as was previously described by Zaytseva et al. [1]. The spectra were registered on an Agilent Cary 300 in the UV-visible-NIR range. The standard spectral indices (Car/Chl and Chl *a*/*b*) were calculated as was previously described [2]. The content of Chlorophyll a, chlorophyll b and the total content of carotenoids were measured (Table S2). All measurements were performed in total of six replicates (three biological replicates with two analytical replicates in each biological one). The data on each estimated parameter were divided into three independent datasets corresponded to studied strains of microalgae. For analysis of the data of absorbance spectroscopy the standard one-way ANOVA (p = 0.99) was applied for the datasets of each parameter for each algal strain. After confirming the hypothesis of differences in certain groups of data, the pairs of samples corresponded to the days of cultivation, were compared by the post-hoc Tukey test. The Kolmogorov-Smirnov test was used to determine normality distribution. The analysis was done in STATISTICA v. 13.3 (StatSoft, Dell, Round Rock, Texas, USA).

Table S2. The contents of main groups of pigments in the cells of three strains of carotenogenic microalgae, *Haematococcus rubicundus* BM7/13, *Bracteacoccus aggregatus* BM5/15, and *Deasonia* sp. NAMSU 934/2 during UV-A treatment. Average values and standard deviations are shown. The data from the same statistical groups for the same microalgal strains are market by same letters.

Time	Chlorophyll *a* (mg/L)	Chlorophyll *b* (mg/L)	Carotenoids (mg/L)
Haematococcus rubicundus BM7/13			
0 d	2.81±0.23 [a]	1.78±0.12 [a]	1.74±0.38 [a]
7 d	0.20±0.04 [b]	0.17±0.02 [b]	0.57±0.12 [b]
14 d	0.21±0.04 [b]	0.20±0.09 [b]	0.92±0.38 [b]
21 d	0.10±0.02 [c]	0.09±0.00 [b]	0.6±0.20 [b]
Bracteacoccus aggregatus BM5/15			
0 d	0.88±0.00 [a]	0.46±0.00 [a]	0.54±0.00 [a]
7 d	0.59±0.12 [b]	0.38±0.010 [a]	0.41±0.06 [a]
14 d	0.89±0.33 [a,b]	0.55±0.19 [a]	0.68±0.25 [a]
21 d	1.02±0.09 [a,b]	Not detected	0.66±0.39 [a,b]
Deasonia sp. NAMSU 934/2			
0 d	1.32±0.28 [a]	0.60±0.14 [a]	0.46±0.10 [a]
7 d	0.89±0.12 [a,b]	0.46±0.06 [a]	1.36±0.07 [b]
14 d	0.46±0.51 [b]	0.53±0.76 [a]	1.10±0.02 [b]
21 d	0.24±0.01 [b]	0.14±0.01 [b]	1.15±0.10 [b]

References

1. Zaytseva, A.; Chekanov, K.; Zaytsev, P.; Bakhareva, D.; Gorelova, O.; Kochkin, D.; Lobakova, E. Sunscreen effect exerted by secondary carotenoids and mycosporine-like amino acids in the aeroterrestrial chlorophyte *Coelastrella rubescens* under high light and UV-A irradiation. *Plants* **2021**, *10*(12), 2601.

2. Chekanov, K.; Litvinov, D.; Fedorenko, T.; Chivkunova, O.; Lobakova, E. Combined production of astaxanthin and β-carotene in a new strain of the microalga *Bracteacoccus aggregatus* BM5/15 (IPPAS C-2045) cultivated in photobioreactor. *Biology* **2021**, *10*(7), 643.
