# Supplementary material for: Differential Responses to UV-A Stress Recorded in Carotenogenic Microalgae Haematococcus rubicundus, Bracteacoccus aggregatus, and Deasonia sp
Source: Plants (Basel). 2022 May 27;11(11):1431. doi: 10.3390/plants11111431 (PMC9183108; doi:10.3390/plants11111431)
Supplement: Supplementary file 1 [file plants-11-01431-s001.zip › suppl file/Supplementary file S3.pdf]

Supplementary S3. Emission spectrum of the light emitting diodes used in the work

The following LEDs were used in the work: 5M 033220 (Arlight, Russia) (spectral range of 380-415 nm, power of 2.9 W/m^2) and cold-white LED UV-A LED COB-X544-8mm 24V White6000 (Arlight, Russia) with the photon flux density of 80 $\mu mol/m^2/s$ (Figure S1). Spectra were registered by an Ocean FX miniature spectrometer (Ocean Optics Inc., Great Britain) equipped with an integrative sphere ISP-80-8-R 80 mm in diameter of the same manufacturer.

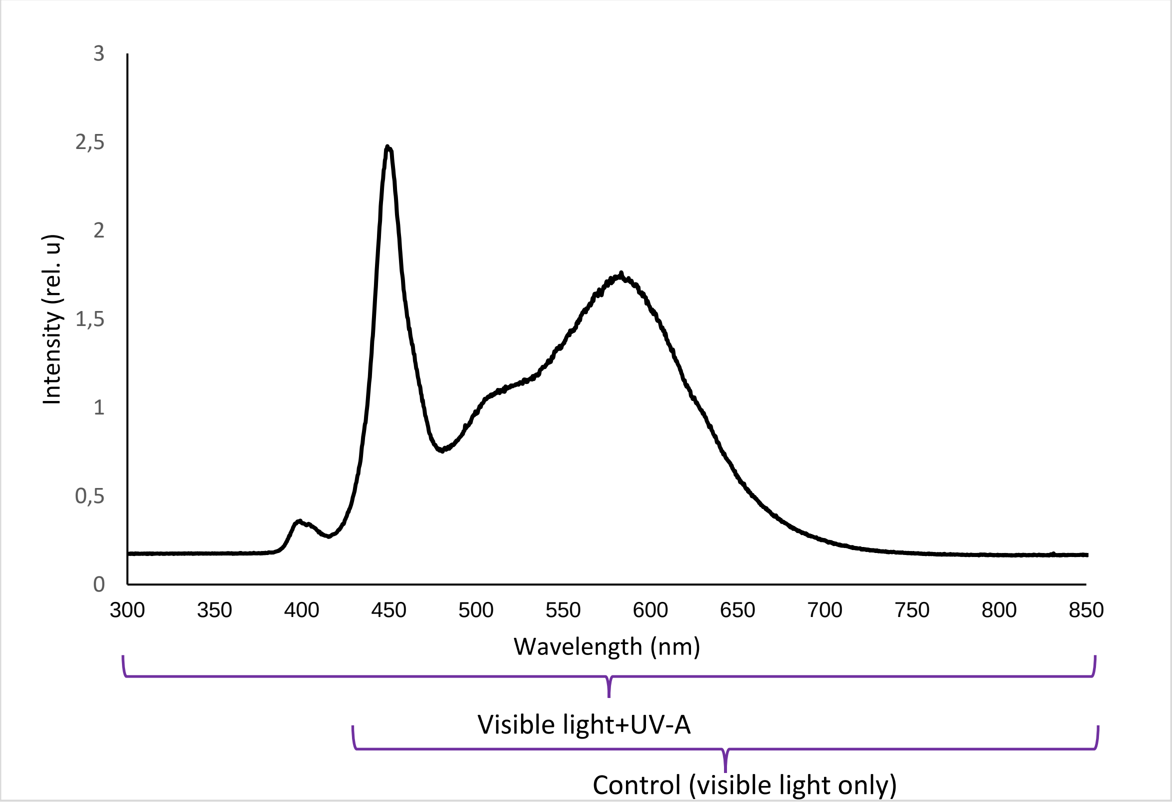

Figure S3. Emission spectrum of LEDs used for culturing of algal cells and sunscreen synthesis induction.
